# Impact of Antibiotic Therapy and Metabolic Parameters in Non-Small Cell Lung Cancer Patients Receiving Checkpoint Inhibitors

**DOI:** 10.3390/jcm10061251

**Published:** 2021-03-17

**Authors:** Angelo Castello, Sabrina Rossi, Luca Toschi, Egesta Lopci

**Affiliations:** 1 Nuclear Medicine Unit, IRCCS-Humanitas Research Hospital, Via Manzoni 56, 20089 Rozzano, Italy; cast.ang@libero.it; 2 Department of Oncology and Hematology, IRCCS-Humanitas Research Center, 20089 Rozzano, Italy; sabrina.rossi@humanitas.it (S.R.); luca.toschi@humanitas.it (L.T.)

**Keywords:** NSCLC, antibiotic therapy, checkpoint inhibitors, immunotherapy, outcomes

## Abstract

Introduction: In the current study, we aimed to assess the impact of antibiotics (ATB) and metabolic parameters on clinical outcome of non-small cell lung carcinoma (NSCLC) patients treated with immune checkpoint inhibitors (ICI). Methods: Data from fifty NSCLC patients referred for ICI between December 2015 and May 2019 were analyzed. All patients underwent 18F-fluorodeoxyglucose positron emission tomography computed tomography (18F-FDG PET/CT) and contrast-enhanced CT at baseline and for response assessment after 6–8 weeks. Patients who received ATB within 1 month before or after the first dose of ICI were compared with those who did not. Response assessment according to iRECIST and EORTC was evaluated, as well as progression-free survival (PFS) and overall survival (OS). For semi-quantitative parameters, we computed metabolic tumor volume (MTV), total lesion glycolysis (TLG) and their variations (∆). Results: Twenty NSCLC cases of 50 (40%) had received ATB. Patients receiving ATB had a higher number of metastases (*p* = 0.046), and were associated with an elevated tumor burden, expressed by TLG (687 vs. 235.3, *p* = 0.007) and MTV (125.6 vs. 40.6, *p* = 0.002), compared to no-ATB patients. According to iRECIST, progressive disease rate was significantly higher for ATB group (64.7% vs. 27.6%, *p* = 0.029). Likewise, PFS was shorter for ATB compared to no-ATB (median 4.1 vs. 12.4 months, *p* = 0.004), while no difference for OS was detected. On multivariate analysis, the effect of ATB remained significant for poor PFS along with performance status (ECOG ≥ 1), and ∆SUVmax. Conclusions: ATB therapy seems to be associated with a worse treatment response, PFS, and higher metabolic tumor burden in NSCLC patients treated with ICI.

## 1. Introduction

One of the major pathways involved in the regulation of adequate immune response in cancer is represented by checkpoints, such as the programmed cell death (PD)-1/programmed cell death ligand-1 (PD-L1) axis, along with anti-CTLA-4 (Cytotoxic T-Lymphocyte Antigen 4) [1,2]. In the last decade, the treatment of several malignancies has significantly changed since the introduction of immune checkpoint inhibitors (ICI). This sort of revolution first started in melanoma patients then was extended to other advanced cancer types [3,4,5]. Notwithstanding this success, response rates with ICI are quite heterogeneous in duration and response, mainly due to the onset of intrinsic resistance [6]. Therefore, there is a stringent need in the medical community to find reliable biomarkers helping the selection of patients mostly benefiting from ICI.

The use of antibiotics (ATB) has undoubtedly represented a milestone in medicine, reducing the morbidity and mortality of several fatal infections. However, the impact of ATB therapy on commensal non-pathogen bacteria has for a long time been unexplored and underestimated [7]. Only recently has the importance of gut microbiota been recognized, a complex milieu where host and millions of microorganisms cohabit and interact in order to shape local and systemic immune response. Indeed, dysregulation of such delicate homeostasis is thought be involved in the pathophysiology of inflammatory bowel disease, allergies, diabetes mellitus, as well in carcinogenesis [8,9,10,11,12]. Various studies have highlighted the importance of a healthy gut microbiota on a positive ICI response [13,14,15,16]. Nevertheless, there are still few data to draw definite conclusions on the deleterious effect of ATB in these patients.

In the current study, we aimed to determine whether antibiotic use during therapy with ICI was associated with clinical variables and metabolic parameters determined on 18F-fluorodeoxyglucose positron emission tomography computed tomography (18F-FDG PET/CT) and how it could affect the treatment outcome.

## 2. Materials and Methods

### 2.1. Study Population

Patients (*n* = 50) with non-small cell lung carcinoma (NSCLC) prospectively enrolled in clinical trials (www.clinicaltrials.gov: NCT03563482 and others) were identified. All patients received either PD-1 inhibitors (nivolumab, pembrolizumab) or PD-L1 inhibitor (atezolizumab) at our institution between December 2015 and May 2019. Patients records were reviewed to determine any ATB therapy within 1 month before and/or after the dose of ICI (ATB vs. no-ATB groups).

Furthermore, all patients underwent to 18F-fluorodeoxyglucose (18F-FDG) positron emission tomography computed tomography (PET/CT), along with contrast-enhanced CT scan performed prior to starting treatment with ICI and repeated every 6–8 weeks during therapy, or as clinically indicated. For the evaluation of tumor response, RECIST 1.1 (Response Evaluation Criteria In Solid Tumors 1.1) for morphologic evaluation, its “immune” update iRECIST, and EORTC (European Organisation for Research and Treatment of Cancer) criteria for metabolic response were used [17,18,19].

The study was conducted in accordance with the Declaration of Helsinki Declaration and approved by local medical ethical board (Prot. Nr. CE Humanitas ex D.M. 8/2/2013 335/17 and 19/17). All patients signed a written informed consent before the enrollment.

### 2.2. Study Endpoints

The primary endpoint was to evaluate the association between ATB therapy and clinical-metabolic variables. Secondary aims were progression-free survival (PFS), defined as the time from the first ICI perfusion to disease progression or death, and overall survival (OS), from the first ICI perfusion to the date of death due to any cause or of censoring at the last time the patient was known to be alive. Median follow-up was 12.4 months (9.7–15.2 months).

### 2.3. 18F-Fluorodeoxyglucose Positron Emission Tomography Computed Tomography (18F-FDG PET/CT) Imaging Protocol

PET/CT images were acquired 60 min after the injection of FDG according to patients’ weight, after a fasting time of approximately 6 h. Acquisitions were performed in EANM Research Ltd. (EARL) accredited scanners: (a) Siemens Biograph LSO 6 scanner (Siemens Erlangen; Munich, Germany), with an integrated 6-slice CT, and (b) GE Discovery PET/CT 690 (General Electric Healthcare; Waukesha, WI, USA), with an integrated 64-slice CT [20]. Image reconstruction and analyses were performed as previously described [21]. Metabolic variables considered for the study comprised the maximum standardized uptake value (SUVmax), defined SUVmax was defined as the value of the highest pixel, average SUV (SUVmean), computed as the mean SUV related to the tumor burden, metabolic tumor volume (MTV) and total lesion glycolysis (TLG = MTV × SUVmean), obtained by applying a SUV threshold of 41%, their variations (∆MTV and ∆TLG).

### 2.4. Statistical Analysis

Differences in the various clinical and metabolic parameters between ATB therapy group and no-ATB were computed using Fisher’s exact test and the Mann–Whitney test as appropriate. Survival curves were estimated by the Kaplan–Meier method and compared with the log-rank test. A multivariable Cox regression model was used to determine hazard ratio (HR) and 95% confidence intervals (95% CI) for PFS and OS between ATB and no-ATB groups, adjusting for other clinic-pathologic features. All statistical analyses were carried out using the Statistical Package for Social Sciences, version 23.0, for Windows (SPSS, Chicago, IL, USA), and *p* values < 0.05 were considered to be statistically significant.

## 3. Results

**Figure 1:**
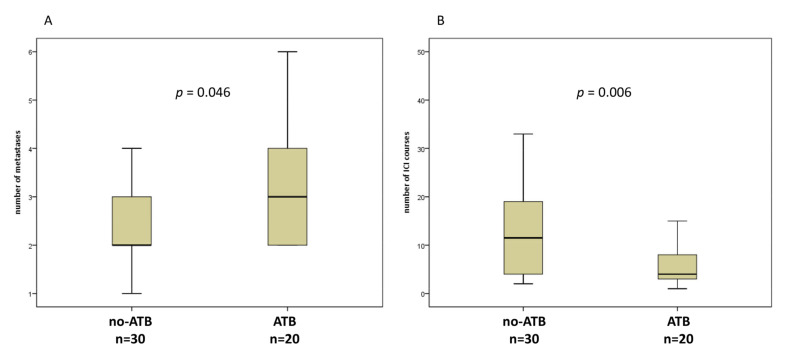
Box plots for number of metastases (**A**) and number of immune checkpoint inhibitors (ICI) courses (**B**) in patients without or with antibiotics (ATB) 1 month before and/or 1 month after the first dose of ICI.

Overall, we enrolled 50 patients (34 male, 16 female, median age 73) with advanced NSCLC and treated with ICI at our hospital. Thirty-one patients (62%) received nivolumab, 16 (32%) pembrolizumab, two patients (4%) with a combination of nivolumab and ipilimumab, and only 1 (2%) patient with atezolizumab. The median number of immunotherapy cycles was 6 (range, 1–47). Twenty (40%) patients received ATB within 1 month before and/or after starting ICI, and 30 (60%) patients did not. Of note, 12 patients received ATB 1 month before, and 11 patients after starting ICI, included 3 patients who received ATB in both interval time. β-Lactams ± inhibitors (*n* = 8) and quinolones (*n* = 8) were the most commonly administered ATB, 1 rifaximin, 1 glycopeptide, whereas 2 were not repeated. The reasons for the use of antibiotics were: pneumonia (*n* = 8), empiric therapy for fever (*n* = 5), urinary tract infections (*n* = 4), and unknown (*n* = 2). The duration of antibiotic use in all cases ranged between 8–14 days. The clinical characteristics of these patients are shown in Table 1. There was no statistical difference between the two groups regarding the age, histology, and peripheral blood biomarkers. However, 13 out of 20 (65%) patients who received ATB therapy had significantly greater extent of disease, expressed by number of metastases > 2 (*p* = 0.046) (Figure 1A). In addition, ATB treated group performed fewer cycles of ICI compared to no-ATB group (4 [1–32] vs. 12 [2–47], *p* = 0.006) (Figure 1B). There were also no statistical differences between the proportion of patients who received ICI as first line therapy or after more lines in the two groups, as well as for PD-L1 score.

### 3.1. Antibiotics (ATB) Therapy and Response Evaluation

**Figure 2:**
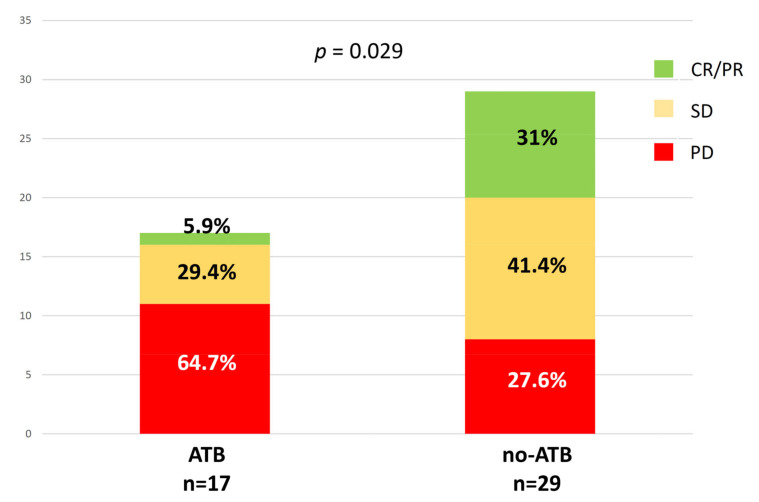
Association between ATB exposure and the radiologic response at the first evaluation according to iRECIST (immunotherapy RECIST) criteria.

Of the 50 patients enrolled, 46 underwent tumor assessment by CT, whereas 18F-FDG PET/CT scans were available for 35 patients at first response assessment. According to iRECIST criteria, we identified the following response categories: complete and partial response (CR, 1 patient and PR, 9 patients, 21.7%), stable disease (SD, 17 patients, 37%), and progressive disease (PD, 19 patients, 41.3%). We found that 9/10 patients with CR/PR has not been treated with ATB in the interval time considered, whereas we observed a statistically significant increase of patients with PD within the ATB group (64.7%), and fewer patients with CR (5.9%), and SD (29.4%) compared to no-ATB group (*p* = 0.029) (Figure 2). On the other hand, EORTC criteria distinguished the following response categories: partial metabolic response (PMR) = 18 patients, stable metabolic disease (SMD) = 9 patients, and progressive metabolic disease (PMD) = 8 patients. However, considering metabolic criteria, no significant difference between ATB and no-ATB groups was found.

### 3.2. ATB Therapy and Metabolic Parameters

**Figure 3:**
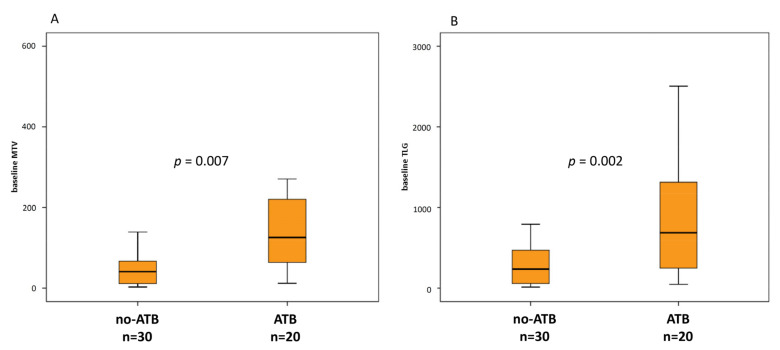
Box plots for metabolic tumor volume (MTV) (**A**) and total lesion glycolysis (TLG) (**B**) at baseline in patients without or with ATB 1 month before and/or 1 month after the first dose of ICI.

**Figure 4:**
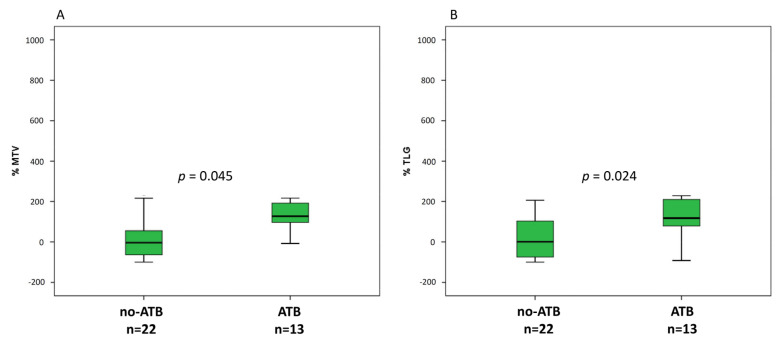
Box plots for number of percentage of variation of MTV (**A**) and TLG (**B**) at first assessment after approximately 6–7 weeks from ICI started.

At baseline, ATB treated patients were associated with greater metabolic tumor burden than no-ATB group, expressed by MTV (189.8 vs. 112.1, *p* = 0.002) and TLG (866.2 vs. 383.5, *p* = 0.007) (Figure 3A,B), whereas there was only a trend for SUVmax (*p* = 0.076). At the first reassessment, after 6–8 weeks according to the ICI administered, ∆TLG and ∆MTV were significantly different between ATB and no-ATB groups. Indeed, median ∆TLG was +117.6% in the ATB group vs. +0.76% in patients who did not take ATB (*p* = 0.045) (Figure 4A). Likewise, median ∆MTV was higher in the ATB-treated patients than no-ATB group (+127.3% vs. −3.7%, *p* = 0.024) (Figure 4B). Distribution of 18F-FDG PET/CT uptake at baseline and at first revaluation is summarized in Table 2.

### 3.3. ATB Therapy and Clinical Outcomes (Progression-Free Survival (PFS) and Overall Survival (OS))

**Figure 5:**
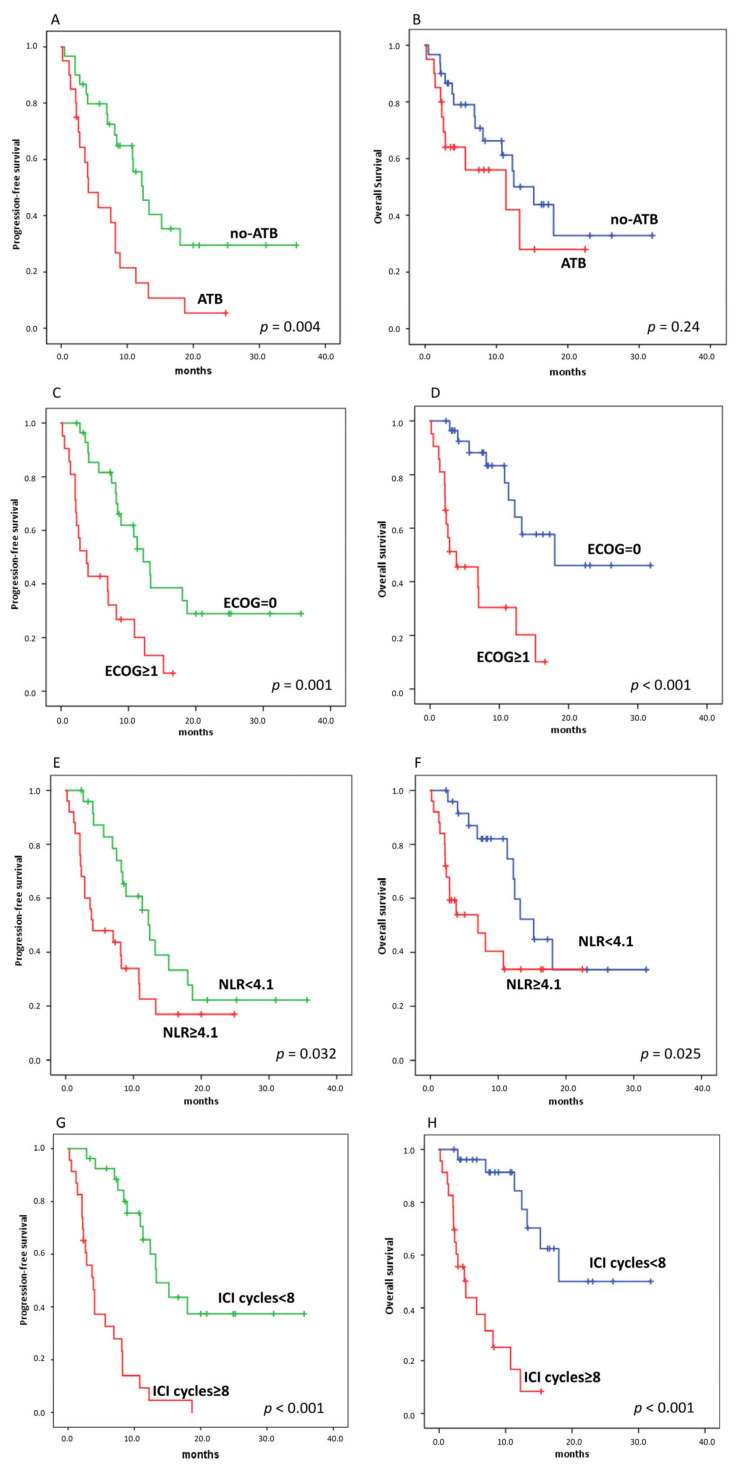
Kaplan–Meier curves for progression-free survival (PFS) (**A**,**C**,**E**,**G**) and overall survival (OS) (**B**,**D**,**F**,**H**), stratifying by using ATB, ECOG (Eastern Cooperative Oncology Group)performance status, NLR, and number of ICI courses, respectively. ((**A**), 12.4 vs. 4.1 months, *p* = 0.004), ((**B**), 11.3 vs. 15.3 months, *p* = 0.24); ((**C**), 12.2 vs. 3.8 months, *p* = 0.001), ((**D**), 18.8 vs. 3.8 months, *p* < 0.001); ((E), 12.4 vs. 4 months, *p* = 0.001), ((**F**), 15.2 vs. 7 months, *p* < 0.025); ((**G**), 3.8 vs. 13.3 months, *p* < 0.001), ((**H**), 4 vs. 18 months, *p* < 0.001).

The median PFS of patients treated with ATB was 4.1 months (95% confidence interval (CI), 1.3–6.9) and the median PFS of patients not treated with ATB was 12.4 months (95% CI, 9.2–15.6) (*p* = 0.004, Figure 5A,B). On the other hand, the median OS between patients treated and those not treated with ATB was not statistically different (median 11.3 vs. 15.3 months, *p* = 0.24). When we considered separately patients who received ATB 1 month before and after ICI started, both PFS and OS were not statistically different.

Among clinical variables, we found that patients with ECOG performance status = 0 and NLR (neutrophil lymphocyte ratio) < 4.1 had longer PFS and OS than patients with ECOG ≥1 and NLR ≥4.1 (Figure 5C–F). Likewise, number of ICI courses above the median value was significantly associated with PFS and OS (Figure 5G,H).

Finally, we carried out a multivariate analysis of the effect of ATB administration, taking into account predictive factors on univariate analysis (Table 3 and Table 4). Both ECOG and ∆SUVmax were significantly associated with worse PFS and OS, while ATB were not associated with OS, but remained significantly associated with worse PFS (HR for ATB 4.2, *p* = 0.004). On the other hand, NLR was predictive only for OS (HR 0.27, *p* = 0.03).

## 4. Discussion

Growing evidence from clinical studies has highlighted the unfavorable effect of ATB in cancer patients treated with ICI. In fact, through the modification of gut microbiota, ATB seem to influence negatively immune response against cancer cells [11]. Indeed, ATB therapy has been linked to shortened PFS, OS, and reduced response rates in patients receiving ICI. However, these observations are still limited and derived mostly from clinical trials [22].

In our study, we reported a meaningful rate of ATB therapy in 20/50 patients (40%) before and/or after starting ICI. Previous studies on ATB treatment have reported quite wide prevalence rates, ranging from 13% to 32% [9]. One of the reason of such large difference may rely on different interval time chosen to define ATB exposition. For example Khan et al. [23] defined ATB use up to 6 months prior ICI, while Tinsley et al. [24] until 6 weeks after ICI initiation. Among the different time intervals used to define ATB therapy in the literature, we have chosen the one comprising between 1 month before and/or 1 month after the first dose of ICI [25]. The rationale for this decision relies on the fact that any microbiome change during ATB will last not less than 1 month and because such criteria have been already used in previous studies, including NSCLC patients [26,27].

Furthermore, our prevalence of ATB use, i.e., 40%, was the highest present in literature. In our work, we had a median age of 75 years, significantly higher than the other studies. Therefore, we speculated that elderly patients could be more susceptible to infections that may justify such elevated ATB use. On the other hand, although in a recent paper Ahmed et al. [28] showed a lower median age for patients treated with antibiotics (52 vs. 66 years), they did not provide any reason behind such evidence.

Among clinical variables, we found patients with ATB medication exposure were significantly associated with a number of metastatic sites >2 at baseline, suggesting that the ATB therapy given to the patients before and/or early during ICI treatment impairs their response to the therapy and favors disease progression. Obviously, it is also possible the contrary, or in other words, patients with extensive disease increase are more susceptible to infections and, therefore, increase the probability of receiving ATB medication. Our results are in line with those of De Rosa et al. [22] who confirmed that tumor burden was significantly higher in patients with renal cell carcinoma treated with ATB prior to ICI. However, our evidence sustained once more the negative effect of ATB on clinical outcomes when patients with antibiotic treatment performed fewer cycles of ICI. Moreover, we also demonstrated a significant association between ATB therapy and morphologic response assessed using iRECIST criteria. Again, our findings are in line with data of Pinato et al. [29] where patients who received ATB had had nearly double the likelihood of poor response to ICI treatment (66 of 151 [44%] vs. 21 of 26 [81%]; *p* = 0.001). On the other hand, although there is a trend for higher NLR median value in the ATB group, this was not significant. In our opinion, such an aspect is mainly due to relative small number of patients in our study.

To the best of our knowledge, this is the first study to investigate the association of 18F-FDG PET/CT metabolic biomarkers with ATB therapy. Of note, we showed that patients in the ATB group had greater median MTV and median TLG values at baseline than those who had not received ATB, which reflect on the imaging plan the number of metastatic sites (i.e., >2) previously mentioned among clinical variables. Our results are in line with those of Guo et al. [30], demonstrated that a large tumor burden, in addition to the use of antibiotics and a high NLR, is a prognostic factor for poor OS in esophageal cancer patients. Therefore, it is likely that patients with a high tumor burden have a larger, but ineffective, inflammatory response. Indeed, as demonstrated by Huang et al. [31], patients who progressed on ICI therapy had wide tumor extension as well as elevated systemic inflammation at baseline. Hence, their hypothesis is that even a robust reinforcement by anti-PD-1 therapy may be clinically ineffective if the tumor burden is massive.

Likewise, percentage change of metabolic tumor burden at the first evaluation, expressed by ∆TMV and ∆TLG, was significantly higher in ATB patients than the other group. Furthermore, we did not provide any difference for baseline SUVmax as well as for response assessment by EORTC criteria, where SUVmax is the only metabolic parameter considered for response. In our opinion, such findings highlight on one hand the SUVmax limit, which does not represent the metabolic status of whole tumor and, on the other hand, the need to incorporate volume-based parameters in the response criteria [32].

Regarding the impact of ATB therapy on ICI efficiency, our results correlated, at least partially, with previous studies [14,22,27]. Routy et al. [14] showed in a large cohort of 249 patients treated with ICI, that early use of ATB (i.e., 2 months before to 1 month after the beginning of ICI) was associated with decreased PFS and OS. Likewise, in another recent study on a cohort of NSCLC patients (*n* = 239), both PFS and OS were shorter for those who received ATB [22]. In our study, we demonstrated a significant reduction in PFS for ATB compared to the no-ATB group in NSCLC patients under ICI treatment, whereas OS was not statistically different between the two groups. In our opinion, lack of power may explain the absence of significant difference on OS, as well as the time interval chosen may affect the distribution of patients in the two groups, then the survival curve.

When we carried out multivariate analysis, we confirmed the negative impact of ECOG performance status, NLR, and metabolic parameter on the outcome with ICI as suggested in our previous work and others papers [33,34,35]. In addition, we also showed that ATB use was associated with poor PFS during ICI (HR = 2.6, CI 95% 1.3–5.0), independently from clinical and metabolic parameters, whereas ATB was not predictive for OS. Previously, two studies have demonstrated that ATB treatment was predictive only for shorter OS in NSCLC patients under ICI therapy [22,36]. Besides the small cohort, also the high number of missing data about PD-L1 status could explain the absence of differences on OS. In fact, there was a trend for patients in the no-ATB group to have higher tumor expression of PD-L1 (i.e., ≥50%, 7 vs. 3 patients), although it is still premature to draw definitive conclusions. By contrast, Kaderbhai et al. [37] did not observe a significant impact of antibiotic medication on PFS under nivolumab, and therefore the authors concluded that microbiota modification induced by ATB does not seem to affect the efficacy of ICI in patients with NSCLC.

Our study has several limitations. This is a monocentric study with a small number of patients, therefore limiting the power of statistical analyses. Our analysis did not take into consideration additional factors with a potential impact on the microbiota composition, e.g., diet or other medications. Moreover, no stool samples were collected in our study for correlation between gut microbiome and metabolic tumor activity. Finally, the lack of valid samples for PD-L1 status examination in most of the patients, as already mentioned, leaves open the possibility that tumor-related features could influence these results.

## 5. Conclusions

In summary, we observed that the use of ATB was associated with a larger tumor burden, expressed by metabolic parameters by 18F-FDG PET/CT and number of metastases, and with worse PFS compared to no-ATB patients during ICI therapy. However, further prospective studies with a larger number of patients are needed to confirm the negative effect of ATB and to develop novel diagnostic tools able to predict response/resistance to ICI.

## Figures and Tables

**Table 1:** Patient characteristics and association between ATB and clinical variables.

	All Patients *n* = 50 (%)	ATB *n* = 20 (%)	no-ATB *n* = 30 (%)	*p*
**Age**				0.77
<73	25 (50)	11 (55)	14 (46.7)	
≥73	25 (50)	9 (45)	16 (53.3)	
**Gender**				0.76
male	34 (68)	13 (65)	21 (70)	
female	16 (32)	7 (35)	9 (30)	
**ECOG performance status**				
0	29 (58)	12 (60)	17 (56.7)	0.82
≥1	21 (42)	8 (40)	13 (43.3)	
**Smoking status**				>0.99
Current/former	44 (88)	26 (86.7)	18 (90)	
None	6 (12)	4 (13.3)	2 (10)	
**Prior lines of treatment**				
**0**	12 (24)	5 (25)	7 (23.3)	0.32
**1**	25 (50)	12 (60)	13 (43.4)	
**2**	11 (22)	3 (15)	8 (26.7)	
**3**	2 (4)	0	2 (6.6)	
**Metastatic sites (median)**				**0.046**
≤2	26 (52)	7 (35)	19 (63.3)	
>2	24 (48)	13 (65)	11 (36.7)	
**Histology**				0.32
Non-squamous	31 (62)	10 (50)	21 (70)	
Squamous others	14 (28) 5 (0.1)	7 (35)	7 (23.3)	
**Tumor PD-L1 status**				0.83
negative	9 (18)	3 (15)	6 (20)	
positive 1–49%	8 (16)	5 (25)	3 (10)	
positive ≥50%	10 (20)	3 (15)	7 (23.3)	
missing	23 (46)	9 (45)	14 (46.7)	
**Cycles of ICI (median)**	8	4	12	
range	(1–47)	(1–32)	(2–47)	**0.006**
**WBC (median)**	7.8	8.5	7.8	
range	(3.8–24.5)	(4.3–24.5)	(3.8–21.3)	0.32
**NLR (median)**	4.1	4.6	4.1	
range	(0.81–32)	(0.81–32)	(1.3–13.2)	0.31
**Platelets (median)**	248	270	248	
range	(118–517)	(144–517)	(118–449)	0.08

Bold: statistically significant *p* values; ECOG: Eastern Cooperative Oncology Group; PD-L1: Programmed Death-Ligand 1.

**Table 2:** Patient characteristics and association between ATB and metabolic variables at baseline and at first re-evaluation.

**Variable**	**All Patients (*n* = 50)**	**ATB (*n* = 20)**	**No-ATB (*n* = 30)**	***p***
SUVmax_baseline	13.7 (4.9–35.7)	16.9 (4.9–35.7)	13.5 (5.3–25.7)	0.076
SUVmean_baseline	5.9 (3.2–10.3)	6 (3.2–9.8)	5.9 (3.4–10.3)	0.572
MTV_baseline	63.7 (2.7–1772)	125.6 (2.7–1772)	40.6 (12–1323.7)	**0.007**
TLG_baseline	330.1 (12.3–2504)	687 (46.5–2504.1)	235.3 (12.3–1645.4)	**0.002**
	**All patients (*n* = 35)**	**ATB (*n* = 13)**	**no-ATB (*n* = 22)**	
∆SUVmax	−16.9 (−100 +144.5)	−18.9 (−61 +144.5)	−8.1 (−100 +107.1)	0.555
∆SUVmean	−1.8 (−100 +130)	−4.1 (−58.2 +130)	0 (−100 +104.9)	>0.99
∆MTV	67.7 (−100 +1245.1)	127.3 (−81.3 +755.5)	−3.7 (−100 +1245.1)	**0.045**
∆TLG	36.5 (−100 +1295.2)	117.6 (−92.1 +505.5)	−0.8 (−100 +1295.2)	**0.024**

Bold: statistically significant *p* values; SUVmax: maximum standardized uptake value; MTV: metabolic tumor volume; TLG: total lesion glycolysis.

**Table 3:** Uni- and multivariate analysis for progression-free survival (PFS).

Parameters	Univariate			Multivariate		
	Hazard Ratio	95% IC	*p* Value	Hazard Ratio	95% IC	*p* Value
Age (<73 vs. ≥73)	1.2	0.6–2.3	0.61	-	-	-
ECOG (0 vs. 1–2)	3.2	1.6–6.4	**0.001**	5.0	1.9–13.7	**0.002**
Number of metastases (≥2)	3.1	1.6–6.1	**0.001**	-	-	-
no-ATB vs. ATB	2.6	1.3–5.0	**0.006**	4.2	1.6–11.3	**0.004**
NLR (<4.1)	0.5	0.25–0.95	**0.04**	-	-	-
SUVmax_baseline (≥13.6)	0.9	0.5–1.8	0.75	-	-	-
SUVmean_baseline (≥5.9)	0.9	1.0–1.7	0.75	-	-	-
TLG_baseline (≥330.1)	1.8	0.9–3.6	0.08	-	-	-
MTV_baseline (≥63.7)	2.5	1.2–4.8	**0.01**	-	-	-
∆SUVmax (<−16.9)	2.9	1.2–6.8	**0.015**	4.2	1.6-10.6	**0.003**
∆SUVmean (<−1.75)	1.9	0.8–4.3	0.13	-	-	-
∆TLG (<67.7)	3.1	1.3–7.4	**0.013**	-	-	-
∆MTV (<36.4)	3.3	1.4–8.0	**0.008**	-	-	-

Bold: statistically significant *p* values; ECOG: Eastern Cooperative Oncology Group.

**Table 4:** Uni- and multivariate analysis for overall survival (OS).

Parameters	Univariate			Multivariate		
	Hazard Ratio	95% IC	*p* Value	Hazard Ratio	95% IC	*p* Value
Age (<73 vs. ≥73)	1.0	0.5–2.3	0.91	-	-	-
ECOG (0 vs. 1–2)	5.1	2.1–12.3	**<0.001**	4.0	1.3–12.1	**0.015**
Number of metastases (≥2)	2.6	1.1–5.9	**0.023**	-	-	-
no-ATB vs. ATB	1.6	0.7–53.7	0.25	-	-	-
NLR (<4.1)	0.4	0.18–0.92	**0.03**	0.3	0.08–0.88	**0.03**
SUVmax_baseline (≥13.6)	0.9	0.4–2.0	0.75	-	-	-
SUVmean_baseline (≥5.9)	0.8	0.4–1.9	0.71	-	-	-
TLG_baseline (≥330.1)	1.5	0.7–3.6	0.27	-	-	-
MTV_baseline (≥63.7)	2.3	1.0–5.3	**0.04**	-	-	-
∆SUVmax (<−16.9)	5.1	1.5–18.1	**0.011**	7.2	1.8–28.6	**0.005**
∆SUVmean (<−1.75)	3.0	0.9–9.3	0.06	-	-	-
∆TLG (<67.7)	2.0	0.7–5.5	0.18	-	-	-
∆MTV (<36.4)	2.8	1–8.2	0.06	-	-	-

Bold: statistically significant *p* values.

## Data Availability

The data presented in this study are available on motivated request to the corresponding author.

## References

[1] Borghaei H., Paz-Ares L., Horn L., Spigel D.R., Steins M., Ready N.E., Chow L.Q., Vokes E.E., Felip E., Holgado E. (2015). Nivolumab versus docetaxel in advanced nonsquamous non-small-cell lung cancer. N. Engl. J. Med..

[2] R Reck M., Rodríguez-Abreu D., Robinson A.G., Hui R., Csőszi T., Fülöp A., Gottfried M., Peled N., Tafreshi A., Cuffe S. (2016). Pembrolizumab versus chemotherapy for PD-L1-positive non-small-cell lung cancer. N. Engl. J. Med..

[3] Robert C., Schachter J., Long G.V., Arance A., Grob J.J., Mortier L., Daud A., Carlino M.S., McNeil C., Lotem M. (2015). Pembrolizumab versus ipilimumab in advanced melanoma. N. Engl. J. Med..

[4] Ferris R.L., Blumenschein G., Fayette J., Guigay J., Colevas A.D., Licitra L., Harrington K., Kasper S., Vokes E.E., Even C. (2016). Nivolumab for Recurrent Squamous-Cell Carcinoma of the Head and Neck. N. Engl. J. Med..

[5] Motzer R.J., Escudier B., McDermott D.F., George S., Hammers H.J., Srinivas S., Tykodi S.S., Sosman J.A., Procopio G., Plimack E.R. (2015). Nivolumab versus everolimus in advanced renal-cell carcinoma. N. Engl. J. Med..

[6] Sharma P., Hu-Lieskovan S., Wargo J.A., Ribas A. (2017). Primary, Adaptive, and Acquired Resistance to Cancer Immunotherapy. Cell.

[7] Zitvogel L., Galluzzi L., Viaud S., Vétizou M., Daillère R., Merad M., Kroemer G. (2015). Cancer and the gut microbiota: An unexpected link. Sci. Transl. Med..

[8] Peterson D.A., Frank D.N., Pace N.R., Gordon J.I. (2008). Metagenomic approaches for defining the pathogenesis of inflammatory bowel diseases. Cell Host Microbe.

[9] Riiser A. (2015). The human microbiome, asthma, and allergy. Allergy Asthma Clin. Immunol..

[10] Upadhyaya S., Banerjee G. (2015). Type 2 diabetes and gut microbiome: At the intersection of known and unknown. Gut Microbes.

[11] Schwabe R.F., Jobin C. (2013). The microbiome and cancer. Nat. Rev. Cancer.

[12] Chaput N., Lepage P., Coutzac C., Soularue E., Le Roux K., Monot C., Boselli L., Routier E., Cassard L., Collins M. (2017). Baseline gut microbiota predicts clinical response and colitis in metastatic melanoma patients treated with ipilimumab. Ann. Oncol..

[13] Vétizou M., Pitt J.M., Daillère R., Lepage P., Waldschmitt N., Flament C., Rusakiewicz S., Routy B., Roberti M.P., Duong C.P. (2015). Anticancer immunotherapy by CTLA-4 blockade relies on the gut microbiota. Science.

[14] Routy B., Le Chatelier E., Derosa L., Duong C., Alou M.T., Daillère R., Fluckiger A., Messaoudene M., Rauber C., Roberti M.P. (2018). Gut microbiome influences efficacy of PD-1-based immunotherapy against epithelial tumors. Science.

[15] Matson V., Fessler J., Bao R., Chongsuwat T., Zha Y., Alegre M.L., Luke J.J., Gajewski T.F. (2018). The commensal microbiome is associated with anti-PD-1 efficacy in metastatic melanoma patients. Science.

[16] Ferris R.L., Blumenschein G., Harrington K., Fayette J., Guigay J., Colevas A.D., Licitra L., Vokes E., Gillison M., Even C. (2017). Abstract CT022: Evaluation of oral microbiome profiling as a response biomarker in squamous cell carcinoma of the head and neck: Analyses from CheckMate 141. Cancer Res..

[17] Eisenhauer E.A., Therasse P., Bogaerts J., Schwartz L.H., Sargent D., Ford R., Dancey J., Arbuck S., Gwyther S., Mooney M. (2009). New response evaluation criteria in solid tumours: Revised RECIST guideline (version 1.1). Eur. J. Cancer.

[18] Seymour L., Bogaerts J., Perrone A., Ford R., Schwartz L.H., Mandrekar S., Lin N.U., Litière S., Dancey J., Chen A. (2017). iRECIST: Guidelines for response criteria for use in trials testing immunotherapeutics. Lancet Oncol..

[19] Young H., Baum R., Cremerius U., Herholz K., Hoekstra O., Lammertsma A.A., Pruim J., Price P. (1999). Measurement of clinical and subclinical tumour response using [18F]-fluorodeoxyglucose and positron emission tomography: Review and 1999 EORTC recommendations. European Organization for Research and Treatment of Cancer (EORTC) PET Study Group. Eur. J. Cancer.

[20] Boellaard R., Delgado-Bolton R., Oyen W.J., Giammarile F., Tatsch K., Eschner W., Verzijlbergen F.J., Barrington S.F., Pike L.C., Weber W.A. (2015). FDG PET/CT: EANM procedure guidelines for tumour imaging: Version 2.0. Eur. J. Nucl. Med. Mol. Imaging.

[21] Castello A., Rossi S., Toschi L., Mazziotti E., Lopci E. (2020). Hyper-progressive Disease in Patients with Non-Small Cell Lung Cancer Treated with Checkpoint Inhibitors: The Role of 18F-FDG PET/CT. J. Nucl. Med..

[22] Derosa L., Hellmann M.D., Spaziano M., Halpenny D., Fidelle M., Rizvi H., Long N., Plodkowski A.J., Arbour K.C., Chaft J.E. (2018). Negative association of antibiotics on clinical activity of immune checkpoint inhibitors in patients with advanced renal cell and non-small-cell lung cancer. Ann. Oncol..

[23] Khan U., Peña C., Brouwer J., Hoffman K., Choudhury A.R., Zhang C., Thakkar P., Betel D., Sarkar S., Sonnenberg G. (2019). Impact of antibiotic use on response to treatment with immune checkpoint inhibitors. J. Clin. Oncol..

[24] Tinsley N., Zhou C., Tan G., Rack S., Lorigan P., Blackhall F., Krebs M., Carter L., Thistlethwaite F., Graham D. (2020). Cumulative Antibiotic Use Significantly Decreases Efficacy of Checkpoint Inhibitors in Patients with Advanced Cancer. Oncologist.

[25] Pinato D.J., Howlett S., Ottaviani D., Urus H., Patel A., Mineo T., Brock C., Power D., Hatcher O., Falconer A. (2019). Antibiotic therapy and outcome from immune-checkpoint inhibitors. J. Immunother. Cancer.

[26] Zhao S., Gao G., Li W., Li X., Zhao C., Jiang T., Jia Y., He Y., Li A., Su C. (2019). Antibiotics are associated with attenuated efficacy of anti-PD-1/PD-L1 therapies in Chinese patients with advanced non-small cell lung cancer. Lung Cancer.

[27] Huemer F., Rinnerthaler G., Westphal T., Hackl H., Hutarew G., Gampenrieder S.P., Weiss L., Greil R. (2018). Impact of antibiotic treatment on immune-checkpoint blockade efficacy in advanced non-squamous non-small cell lung cancer. Oncotarget.

[28] Ahmed J., Kumar A., Parikh K., Anwar A., Knoll B.M., Puccio C., Chun H., Fanucchi M., Lim S.H. (2018). Use of broad-spectrum antibiotics impacts outcome in patients treated with immune checkpoint inhibitors. OncoImmunology.

[29] Pinato D.J., Howlett S., Ottaviani D., Urus H., Patel A., Mineo T., Brock C., Power D., Hatcher O., Falconer A. (2019). Association of prior antibiotic treatment with survival and response to immune checkpoint inhibitor therapy in patients with cancer. JAMA Oncol..

[30] Guo J.C., Lin C.C., Lin C.Y., Hsieh M.S., Kuo H.Y., Lien M.Y., Shao Y.Y., Huang T.C., Hsu C.H. (2019). Neutrophil-to-lymphocyte Ratio and Use of Antibiotics Associated with Prognosis in Esophageal Squamous Cell Carcinoma Patients Receiving Immune Checkpoint Inhibitors. Anticancer Res..

[31] Huang A.C., Postow M.A., Orlowski R.J., Mick R., Bengsch B., Manne S., Xu W., Harmon S., Giles J.R., Wenz B. (2017). T-cell invigoration to tumour burden ratio associated with anti-PD-1 response. Nature.

[32] Cheng G., Huang H. (2018). Prognostic Value of 18F-Fluorodeoxyglucose PET/Computed Tomography in Non-Small-Cell Lung Cancer. PET Clin..

[33] Dumenil C., Massiani M.A., Dumoulin J., Giraud V., Labrune S., Chinet T., Giroux Leprieur E. (2018). Clinical factors associated with early progression and grade 3-4 toxicity in patients with advanced non-small-cell lung cancers treated with nivolumab. PLoS ONE.

[34] Diem S., Schmid S., Krapf M., Flatz L., Born D., Jochum W., Templeton A.J., Fueh M. (2017). Neutrophil-to-Lymphocyte ratio (NLR) and Platelet-to-Lymphocyte ratio (PLR) as prognostic markers in patients with non-small cell lung cancer (NSCLC) treated with nivolumab. Lung Cancer.

[35] Castello A., Toschi L., Rossi S., Mazziotti E., Lopci E. (2020). The immune-metabolic-prognostic index and clinical outcomes in patients with non-small cell lung carcinoma under checkpoint inhibitors. J. Cancer Res. Clin. Oncol..

[36] Ouaknine Krief J., Helly de Tauriers P., Dumenil C., Neveux N., Dumoulin J., Giraud V., Labrune S., Tisserand J., Julie C., Emile J.F. (2019). Role of antibiotic use, plasma citrulline and blood microbiome in advanced non-small cell lung cancer patients treated with nivolumab. J. Immunother. Cancer.

[37] Kaderbhai C., Richard C., Fumet J.D., Aarnink A., Foucher P., Coudert B., Favier L., Lagrange A., Limagne E., Boidot R. (2017). Antibiotic Use Does Not Appear to Influence Response to Nivolumab. Anticancer Res..

